# Selective effects of Δ^9^-tetrahydrocannabinol on medium spiny neurons in the striatum

**DOI:** 10.1371/journal.pone.0200950

**Published:** 2018-07-26

**Authors:** Mónica R. Fernández-Cabrera, Alejandro Higuera-Matas, Isabel Fernaud-Espinosa, Javier DeFelipe, Emilio Ambrosio, Miguel Miguéns

**Affiliations:** 1 Departamento de Psicobiología, Facultad de Psicología, Universidad Nacional de Educación a Distancia (UNED), Madrid, Spain; 2 Laboratorio Cajal de Circuitos Corticales, Centro de Tecnología Biomédica, Universidad Politécnica de Madrid (UPM), Madrid, Spain; 3 Instituto Cajal, Consejo Superior de Investigaciones Científicas, Madrid, Spain; 4 Centro de Investigación Biomédica en Red sobre Enfermedades Neurodegenerativas (CIBERNED), Madrid, Spain; 5 Departamento de Psicología Básica I, Facultad de Psicología, Universidad Nacional de Educación a Distancia (UNED), Madrid, Spain; Universidade Federal do ABC, BRAZIL

## Abstract

Derivatives from the *Cannabis* plant are the most commonly abused illegal substances in the world. The main psychoactive component found in the plant, Δ-9-tetrahydrocannabinol (THC), exerts its effects through the endocannabinoid system. Manipulations of this system affect some types of learning that seem to be dependent on dorsal striatum synaptic plasticity. Dendritic spines exhibit important synaptic functional attributes and a potential for plasticity, which is thought to mediate long-lasting changes in behaviour. To study the possible structural plasticity changes that prolonged THC administration might exert in the dorsal striatum, adult, male C57BL6/J mice were intraperitoneally injected with THC (10mg/kg) or vehicle for 15 days followed by a 7-day drug-free period. Using single cell intracellular injections of Lucifer Yellow, confocal microscopy, and 3D reconstruction of labelled neurons, we studied dendritic spine density and spine size in medium spiny neurons (MSNs) of the anterior dorsolateral striatum (aDLS) and posterior dorsomedial striatum (pDMS). We found that the THC treatment increased dendritic spine density in the distal part of the dendrites of MSNs in the pDMS, but no changes were found in the rest of the parameters analysed in either region studied. We also observed that dendritic spines of MSNs of pDMS presented lower volume and surface area values than MSNs of the aDLS. These results seem to indicate that THC could induce structural plasticity alterations in the circuits involving pDMS MSNs.

## Introduction

Cannabis is the most commonly abused illegal substance in the world [1,2]. The principal psychoactive effects of this drug are mainly due to the pharmacological effects induced by Δ^9^-tetrahydrocannabinol (THC) through the specific activation of CB1 cannabinoid receptors [3–5]. These receptors, which are principally found at presynaptic terminals, modulate excitatory and inhibitory neurotransmitter delivery, usually inhibiting their release [6–8]. The involvement of CB1 cannabinoid receptors in THC addictive properties has been shown in several studies (for a review, see [9]).

It is proposed that addiction could be related to the transition from a free-choice behaviour to a progressive loss of volitional control over reward-seeking, causing a rapid development of response habits and eventually compulsive behaviour [10]. The dorsal striatum is involved in the anatomical circuits that support goal-oriented behaviour and habit formation. The dorsolateral region has been linked to habit formation, whereas the dorsomedial region seems to play a role in goal-oriented behaviour [11–14]. Studies with some tasks to evaluate these kinds of behaviours demonstrate that lesions in the posterior region of dorsomedial striatum (pDMS) are more important in instrumental learning than the more anterior region [12], and it is proposed that goal-directed response acquisition depends also primarily on plasticity in the pDMS [15,16]. Moreover, a recent study reports that prelimbic cortex and pDMS connection is required to acquire response–outcome value [17]. Conversely, the anterior dorsolateral region of the striatum (aDLS) is primarily involved in habit formation [18–20,13]. Thus, focusing on these regions of the dorsal striatum could provide relevant information about the role of cannabinoids in the transition between goal-directed and habitual behaviours. Furthermore, CB1 receptors are widely distributed throughout the brain, and follow a distinctive lateral-medial gradient expression in the dorsal striatum [21–24]. CB1 knockout mice displayed a decreased predisposition for habit formation and acute pharmacological blockade of CB1 receptors induced similar behavioural patterns, indicating that endocannabinoid signalling through CB1 receptors is necessary for habit formation [25].

Experience-dependent changes are thought to be mediated by the reorganization of synaptic connections in neural circuits (structural plasticity) that support a given behaviour [26,27]. Exposure to drugs of abuse produce persistent modifications in neuronal morphology (for a review see [28,29]), facilitating the experience-dependent changes in specific neural circuits that promote addiction. Not surprisingly, drug addiction has been suggested to be a pathology of the brain mechanisms mediating neuroplastic adaptations [30,31]. Repeated exposure to cannabinoids affect some types of learning that seem to be dependent on the dorsal striatum [32] and could play an important role in the circuits involved in addiction [10]. Moreover, THC administration has been associated with (i) sensitization; (ii) the motivational properties of withdrawal; (iii) cognitive impairment; and (iv) morphological modifications of the dendrites and dendritic spines (for simplicity, spines) [33–37].

It has been shown that different portions of the dendrites of medium spiny neurons (MSNs) receive synaptic connections from distinct brain areas. Inputs to the cell body and proximal dendrites are from GABAergic parvalbumin and cholinergic striatal interneurons, whereas inputs to the distal part of the dendrites are from the cerebral cortex, nigrostriatal dopamine (DA) afferents, and thalamus [38]. Given the specific distribution of these inputs, knowing the exact location of drug-induced changes in spine density and morphology in different portions of the dendritic tree is of interest. Attention has also been paid to functional differences between these dendritic compartments [39,40]. For example, in long-term potentiation induction in the hippocampus, proximal dendrites are related to temporal integration of synaptic inputs, while distal spines selectively facilitated coincidence detection [41]. Distal spines have also been associated with prolonged “up states” in striatal MSNs [42]. Likewise, this dichotomy has also been previously reported as drug-induced changes in different dendritic compartments [43,44].

Repeated administration of THC reduces CB1 receptor expression [45] and impairs LTD and synaptic depotentiation in the dorsal striatum [46]. However, to date, no studies have been performed to analyse the potential changes in dendritic spine density and dendritic spine morphology in dorsal striatum induced by prolonged administration of THC. Therefore, the purpose of the present study was to analyse in detail the effects of prolonged THC treatment to assess possible morphological changes in MSN dendrites and dendritic spines of the anterior dorsolateral striatum (aDLS) and posterior dorsomedial striatum (pDMS)—two areas that, as mentioned above, are related to the transition from a volitional behaviour to habit formation. Moreover, we have also analysed the possible underlying differences between the morphological features of pDMS and aDLS MSNs spines.

## Material and methods

### Subjects

Animal work have been conducted in accordance with the European Union guidelines for the care of laboratory animals (Directive 2010/63/EU). Mice were deeply anesthetized with chloral hydrate (16%, 0.4 g/kg) previous to intracardially perfusion. The Bioethical Committee of the Universidad Nacional de Educación a Distancia (UNED) approved this research.

Adult male C57BL6/J mice (Charles River Laboratories International, Inc.), 7–8 weeks old at the beginning of the experiments, were used in this study (n = 20). Animals were experimentally naïve and, unless otherwise specified, they had free access to MLab Rodent Table feed (CIBERTEC, Madrid, Spain) and tap water. They were housed in pairs in a climate-controlled room (23°C) with a 12-h light–dark cycle (08:00–20:00 lights on).

### THC treatment

THC (Dronabinol, THC Pharm Gmbh, Germany) was diluted in ethanol, partitioned into smaller aliquots (10 mg/0.5 ml) and stored at– 20°C. When needed, the THC aliquots were prepared in a vehicle of absolute ethanol, cremophor (Cremophor EL, Polyoxyl 35 hydrogenated castor oil; Sigma-Aldrich Chemical, Madrid, Spain), and saline at a ratio of 1:1:18, and the volume for both vehicle and drug injection was 10 ml/kg of body weight. The ethanol concentration in the THC solution and in the vehicle (VH) was less than 5%, resulting in ethanol doses of approximately 0.05–0.15 g/kg. Mice were randomly assigned to two groups (THC and VH, n = 10 in each group) and were given i.p. injections of either THC (10 mg/kg) or the vehicle solution once daily for 15 days. This dose was chosen based on data from a review of the literature [46–49]. A 7-day drug-free interval prior to tissue processing was used in order to allow clearance of THC and its metabolites.

### Tissue processing

Animals were anaesthetized by an intraperitoneal injection of 16% chloral hydrate (0.40 g/kg), and intracardially perfused with 50 ml of 1% paraformaldehyde in 0.1 M phosphate buffer (PB; pH 7.3) followed by 100 ml of 4% paraformaldehyde and 0.125% glutaraldehyde in 0.1 M PB (pH 7.3), at a flow rate of 5 ml/min. Immediately after perfusion, brains were removed and post-fixed for 6 h at room temperature in the same fixative. After post-fixation, 150-μm horizontal vibratome (Lancer 1000; St Louis, MO, USA) sections that included the regions of interest were obtained.

### Lucifer yellow intracellular injections

**Fig 1:**
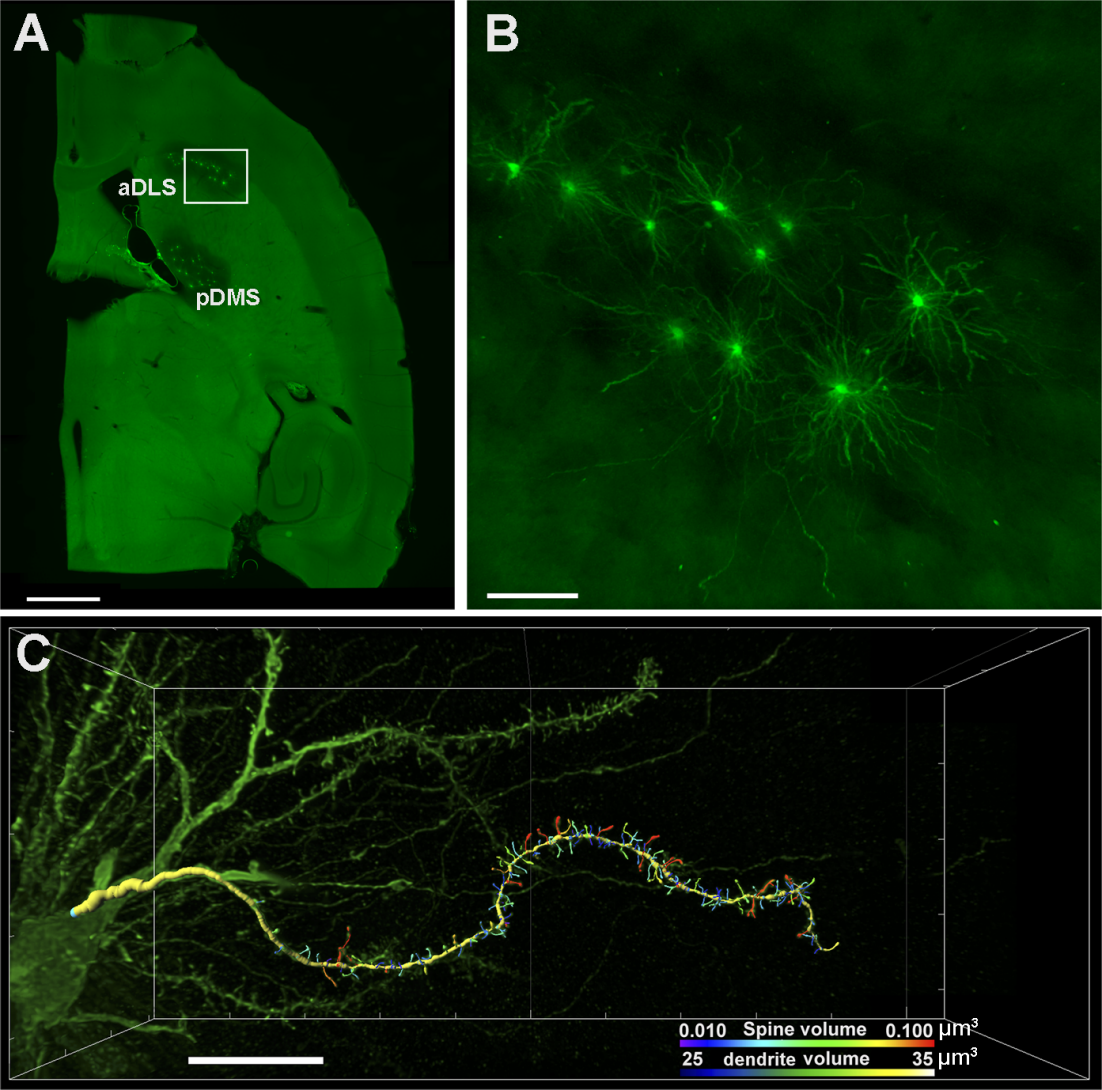
Dorsal striatum MSN 3D dendritic spine reconstruction. (A) Fluorescence panoramic image of a horizontal encephalic section showing anterior dorsolateral striatum (aDLS) and posterior dorsomedial striatum (pDMS) neurons injected with LY. (B) Confocal microscopy projection showing a higher magnification of aDLS MSNs boxed in A. (C) 3D reconstruction of dendrite and spines using Imaris Filament Tracer software (Imaris 7.6, Bitplane AG, Zurich, Switzerland). Bars represent 500 μm in A, 100 μm in B, and 10 μm in C.

Single-cell intracellular injections were performed according to the methods described in [50] with some modifications. Each slice was labelled with 4’, 6-diamidino-2-phenylindole (DAPI, D9542; Sigma, St Louis, MO, USA). On horizontal slices, all sample cells (Fig 1A) were located between Interaural: 6.76; Bregma: -3.84 and Interaural: 4.96; Bregma: -5.04 following the atlas available at The Mouse Brain Library [51]. A continuous current (1–10 nA) was used to inject cells with the fluorescent dye Lucifer Yellow [LY; 8% in 0.1 M Tris buffer (pH 7.4)] and at least 15 anterior dorsolateral striatum (aDLS) and 15 posterior dorsomedial striatum (pDMS) medium spiny neurons per animal were impaled (Fig 1A and 1B). To visualize the morphology of the intracellularly injected cells, sections were processed with a rabbit antibody against LY (1:400.000 generated at the Cajal Institute) diluted in stock solution (2% bovine serum albumin, 1% Triton X-100, and 5% sucrose in PB). Subsequently, they were incubated in a biotinylated goat anti-rabbit IgG (Amhersham; 1:1000) and finally revealed with a streptavidin-Alexa fluor 488-conjugate (1:1000; Invitrogen, Carlsbad, CA, USA). Sections were then mounted and cover-slipped using ProLong® Gold antifade reagent (Invitrogen, CA, USA) to preserve the tissue.

### Morphometric analysis of the dendrites and dendritic spines

For dendrite reconstruction, z-Stack images (8–10 entire dendrites per animal, Fig 1B) were taken for analysis with a Zeiss confocal microscope (LSM 710, equipped with an Axio Observer Z1 inverted microscope; Carl Zeiss MicroImaging GmbH, Germany), using a 0.082 × 0.082 × 0.080 μm^3^ voxel size with a x63 immersion objective (Zeiss Objective Plan-Apochromat 63x/1.40 NA Oil DIC M27). The image stacks were stitched into a single volume dataset using the image stitching plugins in Fiji software [52]. They were then deconvoluted using Autodeblur software (MediaCybernetics, Inc., Bethesda, MD, USA) to diminish the blur around spines. Z-stacks were reconstructed using Imaris software (version 7.6.5, Bitplane Inc., St. Paul, MN), and dendritic shafts and spines were auto-detected in the xy plane using the AutoDepth mode in Imaris FilamentTracer (Imaris, Bitplane, Inc.; Fig 1C). This module opens an image stack of confocal images and allows 3D reconstruction and measurement of different morphological parameters of the dendritic shaft and spines. We analysed: dendritic size (mean diameter, surface area, volume and length) and spine size (area, volume and length), as provided by Imaris software. Dendritic spine density was calculated by dividing the total number of spines per dendrite by total dendritic length.

Dividing the dendrite into three identical segments lengthwise, the proximal dendrite was defined as the closest third to the soma and the distal dendrite was defined as the furthest third from the soma.

### Statistical analysis of the data

One animal from the vehicle group was lost during perfusion, and three animals—one from the vehicle group and two from the THC group—were discarded as a consequence of the poor quality of the tissue fixation. Thus, the final analysis of the data was performed with 8 animals for each condition. To explore the differences in dendrite and spine size parameters between treatments, a multivariate analysis of variance (MANOVA; Wilks’ Lambda) was used with *treatment* as the predictor variable and the dependent variables *diameter*, *surface area* and *volume* for dendrites, and *density*, *area*, *volume* and *length* for spines, measured in the aDLS and pDMS. To compare variations in spine parameters between regions, a MANOVA was used with *region* as the predictor variable and *spine density*, *area volume and length* as dependent variables. When significant, the MANOVA was followed up with the Bonferroni post hoc. To analyse differences between proximal and distal spines, a 2-way mixed analysis of variance (ANOVA) test was used with *treatment* and *region* as independent variables and *part of the dendrite* as within-subject variable. Post hoc comparisons were carried out using pair-wise comparisons with a Bonferroni correction for p values. Cumulative frequencies were compared using the Kolmogorov–Smirnov (K–S) test. All statistical analyses were performed using the SPSS statistical package (version 24.0). The partial eta squared (η^2^_p_) effect size—as provided by the SPSS package—was used and the level of significance was set at α = 0.05.

## Results

### THC effects on dendritic size

**Fig 2:**
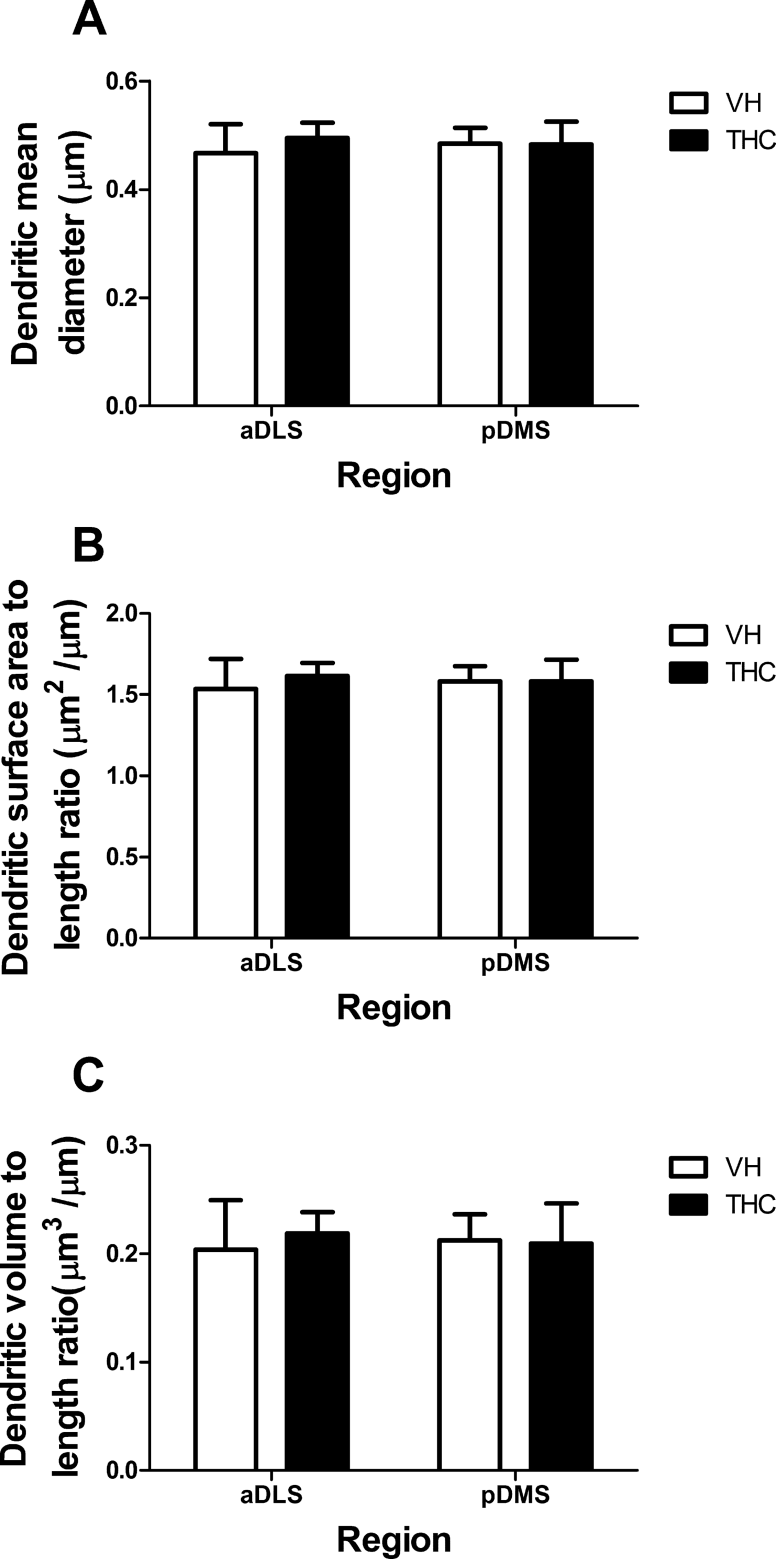
THC effects on dendritic size. Dendritic size parameters are depicted for VH- and THC-treated animals (n = 8 per group) in aDLS and pDMS MSNs: dendritic mean diameter (A), dendritic surface area to length ratio (B), and dendritic volume to length ratio (C). Data is presented as mean ± s.e.m.

In the case of both pDMS and aDLS MSN dendrites, no significant differences were found between THC-treated and VH control subjects regarding any of the morphometric parameters for dendrites (dendritic mean diameter, dendritic volume and dendritic surface area; the latter two parameters normalized to dendritic length (Fig 2; S1 Fig).

No differences between pDMS and aDLS MSN dendrites were found in the parameters related to dendritic size.

### Regional and dendritic compartment-specific effects of THC on dendritic spine density

**Fig 3:**
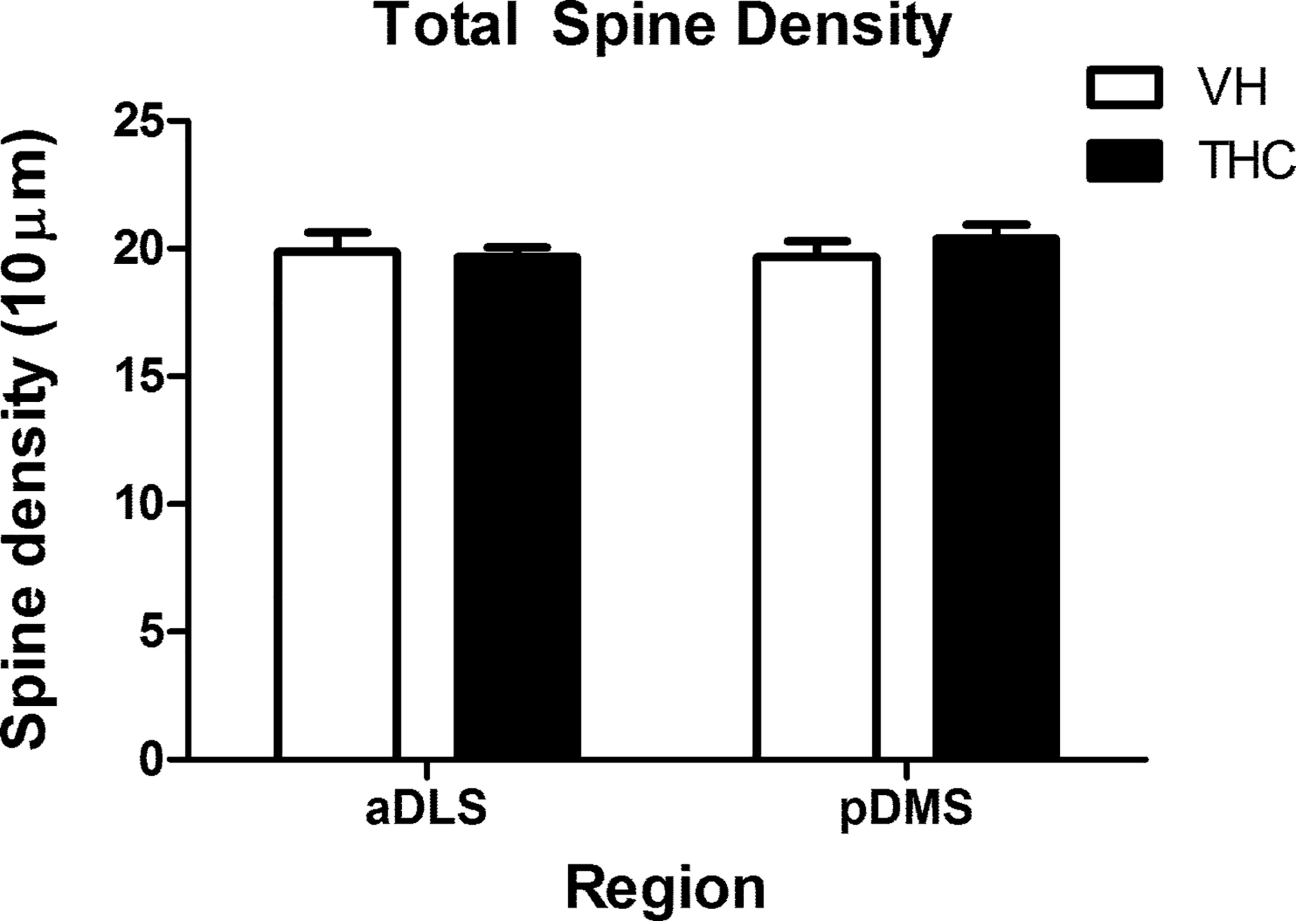
Dendritic spine density in the whole dendrite. The picture shows dendritic spine density analysed in the dendrites as a whole for VH- and THC-treated animals (n = 8 per group) in aDLS and pDMS MSN dendrites. No statistically significant differences were observed. Data is presented as the mean ± s.e.m.

Spine densities in the whole dendrite and in the proximal and distal compartments were quantified. When analysed as a whole, no statistically significant differences between the dendrites of VH- and THC-treated animals were observed. Furthermore, there were no differences in spine densities between pDMS and aDLS MSNs, regardless of the treatment applied (Fig 3; S2 Fig).

**Fig 4:**
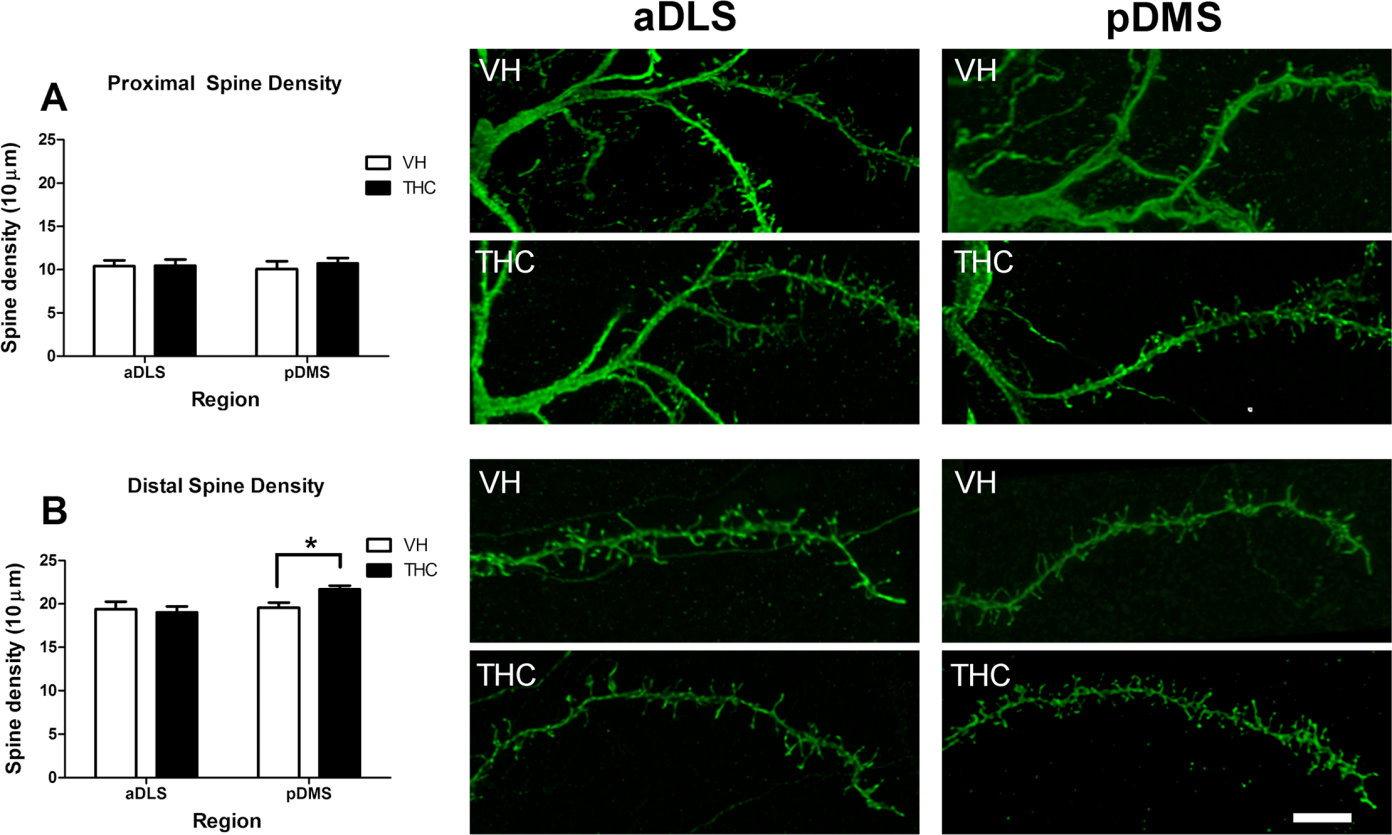
Dendritic spine density in proximal and distal compartments. The picture illustrates dendritic spine densities in the proximal (A), and distal (B) parts for VH- and THC-treated animals (n = 8 per group) in aDLS and pDMS MSN dendrites. Proximal (top) and distal (bottom) dendrite images in each region and condition are shown. An increase in the number of spines in THC compared to the VH mice in the distal part of the dendrite in the pDMS can be observed. Bar represents 5 μm. Data is presented as the mean ± s.e.m.; *p <0.05.

THC treatment had no effects on spine density in the proximal part of MSN dendrites in any of the analysed regions (Fig 4A; S2 Fig). This portion of the dendrite is almost unspiny at 10 μm from the soma (1.32 ± 0.30 spines per 10 μm in pDMS and 1.24 ± 0.22 spines per 10 μm in aDLS) and it reaches distal values at 40 μm (24.53 ± 1.26 spines per 10 μm in pDMS and 24.25 ± 1.41 spines per 10 μm in aDLS). Interestingly, the compartment analysis of spine density showed a statistically significant effect of *treatment* (F_2, 12_ = 4.415, p = 0.037, η^2^_p_ = 0.424) in the distal part of the MSN dendrites. The post hoc analysis showed that THC administration increased spine density in this portion of the dendrites in the pDMS (F_1, 13_ = 8.643, p = 0.011) but not in the aDLS (Fig 4B; S2 Fig).

No differences between regions were found in the proximal or distal part of the dendrite.

### Regional and dendritic compartment-specific effects of THC on spine size

**Fig 5:**
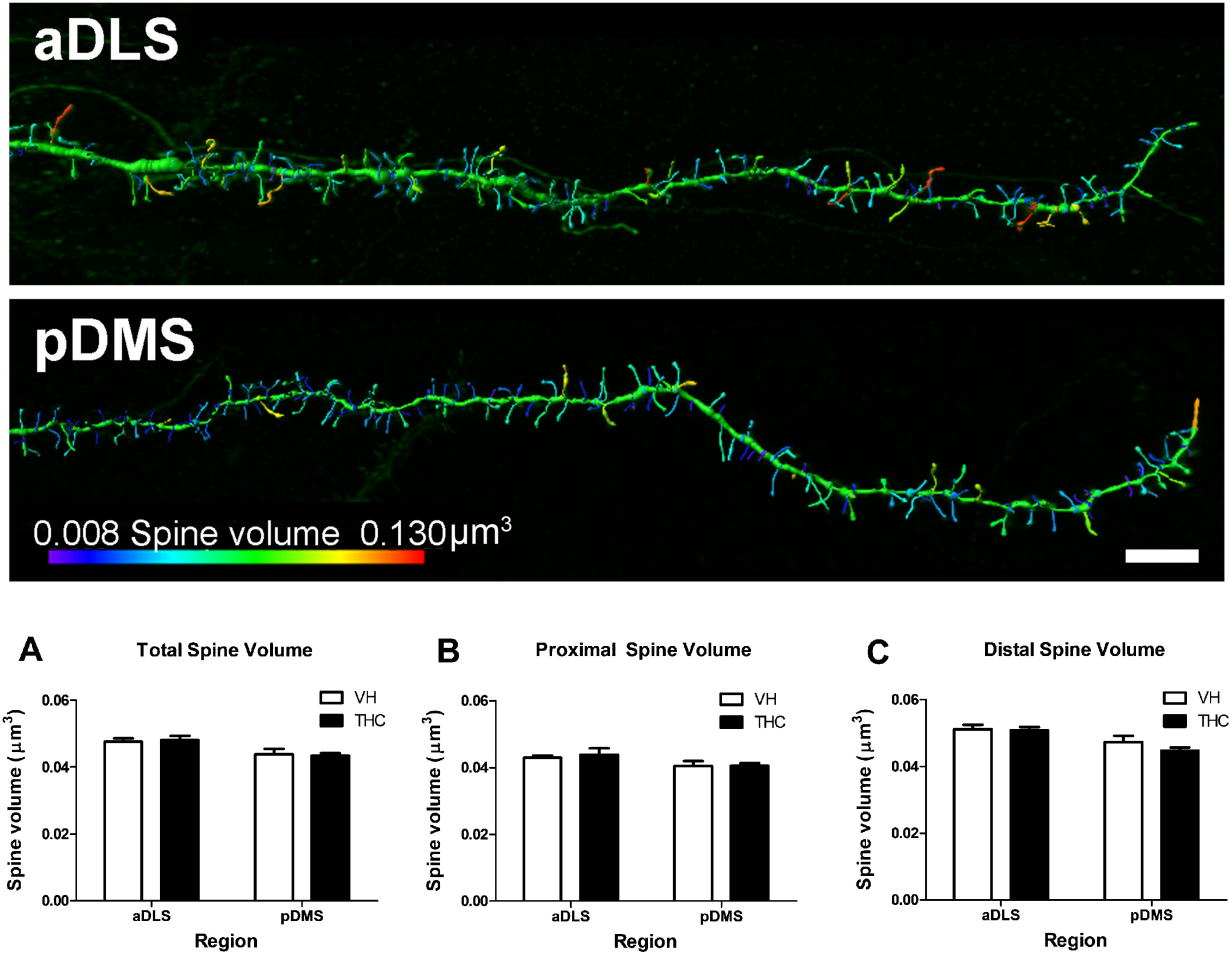
Dendritic spine volume. Spine volume averaged values for the whole dendrite (A), and for proximal (B) and distal (C) parts for THC- and VH-treated animals (n = 8 per group) in aDLS and pDMS MSN dendrites. THC did not affect spine volume but the underlying spine volume was observed to be lower in the pDMS spines compared to the aDLS for the total dendrite and for the proximal and distal parts. A comparison of spine size between aDLS (top) and pDMS (bottom) dendrites in control animals is shown. The pseudo-colour image shows that pDMS has a higher percentage of small spines (lower volume, coloured in blue) than aDLS. Bar represents 5 μm. Data are presented as mean ± s.e.m.

**Fig 6:**
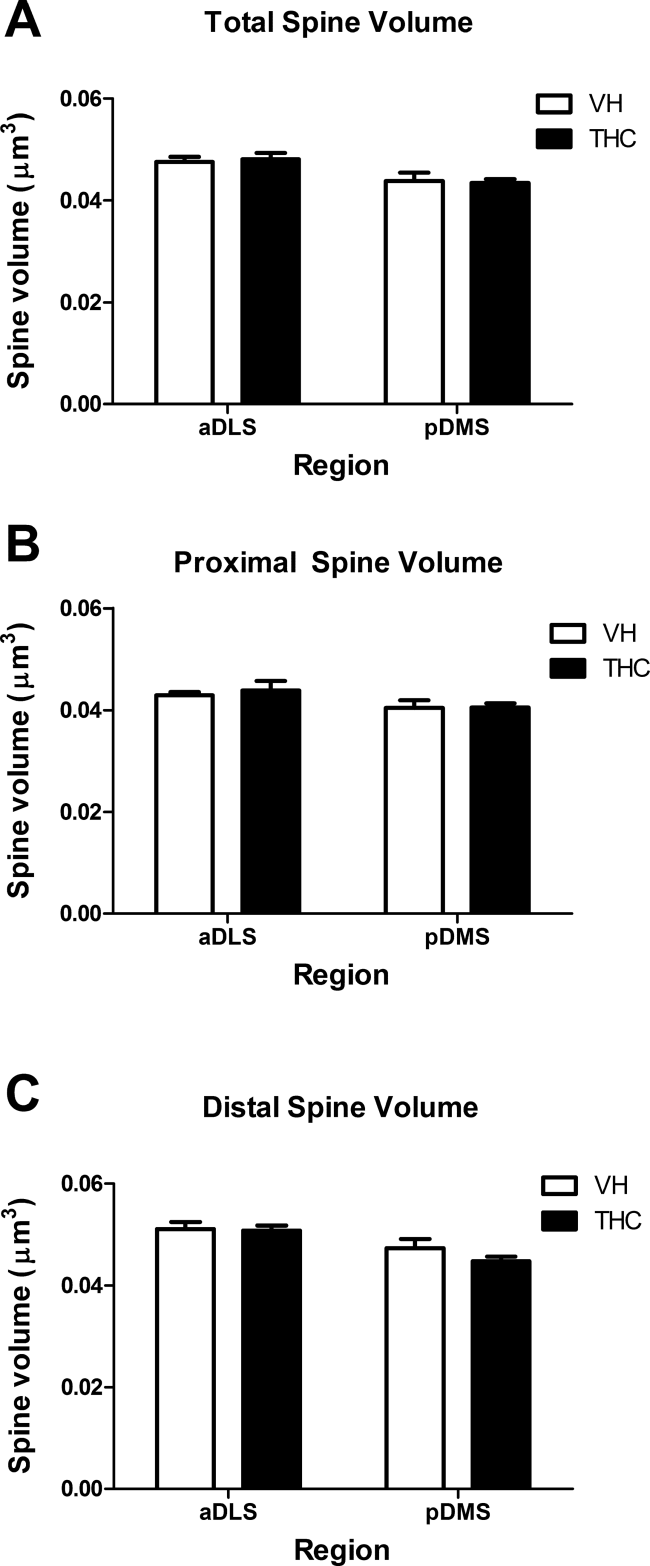
Dendritic spine area. Spine area averaged values for the whole dendrite (A), and for proximal (B) and distal (C) parts for THC- and VH-treated animals (n = 8 per group) in aDLS and pDMS MSN dendrites. THC did not affect the spine area but the underlying spine area was lower in pDMS neurons compared to aDLS in the total dendrite and the distal part. Data are presented as mean ± s.e.m.

**Fig 7:**
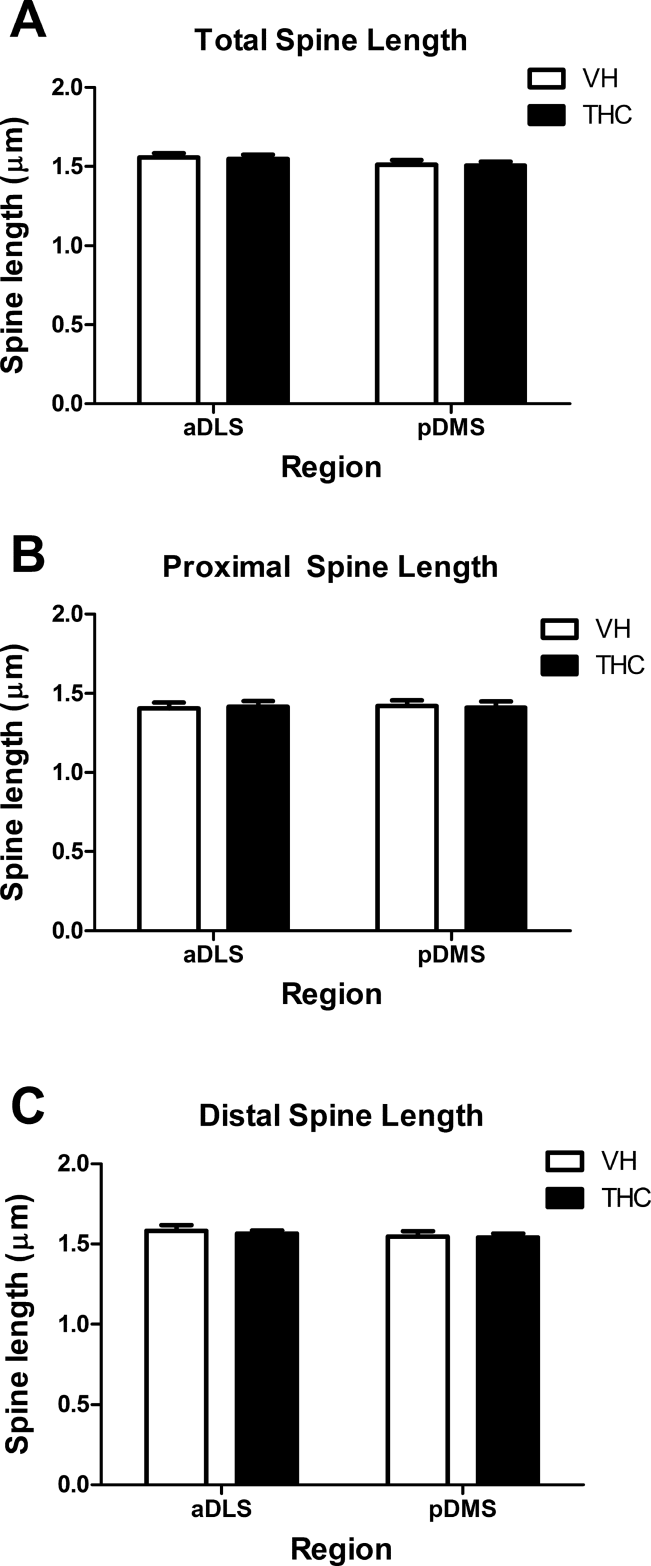
Dendritic spine length. Spine length averaged values for the whole dendrite (A), and for the proximal (B) and distal (C) parts for THC- and VH-treated animals (n = 8 per group) in aDLS and pDMS MSN dendrites. THC did not seem to affect spine length and no differences were observed in pDMS neurons with respect to aDLS. Data are presented as mean ± s.e.m.

THC did not affect spine volume, area, or length in the dendrites analysed as a whole for either aDLS or pDMS MSNs. The compartment analysis also showed no differences in any of these parameters between treatments in the proximal or the distal part of aDLS or pDMS dendrites (Figs 5, 6 and 7; S3 Fig).

However, the MANOVA showed an effect of *region* for the whole dendrite (F_4,26_ = 3.671, p = 0.017, η^2^_p_ = 0.361). The post hoc analysis revealed that aDLS spines had greater volume (F_1,29_ = 8.694, p = 0.001) and larger spine area (F_1,29_ = 13.018, p = 0.006) than pDMS MSN spines, but no differences were observed in spine length (Figs 5A, 6A and 7A, respectively). As for the compartment analysis, in the distal parts we also observed an effect of *region* (F_4, 25_ = 3.809, p = 0.015, η^2^_p_ = 0.379); spines in aDLS dendrites had a larger area (F_1,28_ = 13.750, p = 0.011) and greater volume (F_1,28_ = 7.368, p = 0.001) than pDMS dendrites. We did not find differences in spine length in this case either (Figs 5C, 6C and 7C).

No *region* effect was observed at proximal dendrites (Figs 5B, 6B and 7B).

Regardless of the region or treatment applied, spines in the proximal portion were smaller than in distal dendrite. Proximal spines had less surface area, volume and length than distal spines (F_1,27_ = 69.210, p < 0.0001, η^2^_p_ = 0.719; F_1,26_ = 63.249, p < 0.0001, η^2^_p_ = 0.709; F_1,28_ = 60.877, p < 0.0001, η^2^_p_ = 0.685, respectively).

### Underlying differences in spine size frequency distributions between pDMS and aDLS MSNs

**Fig 8:**
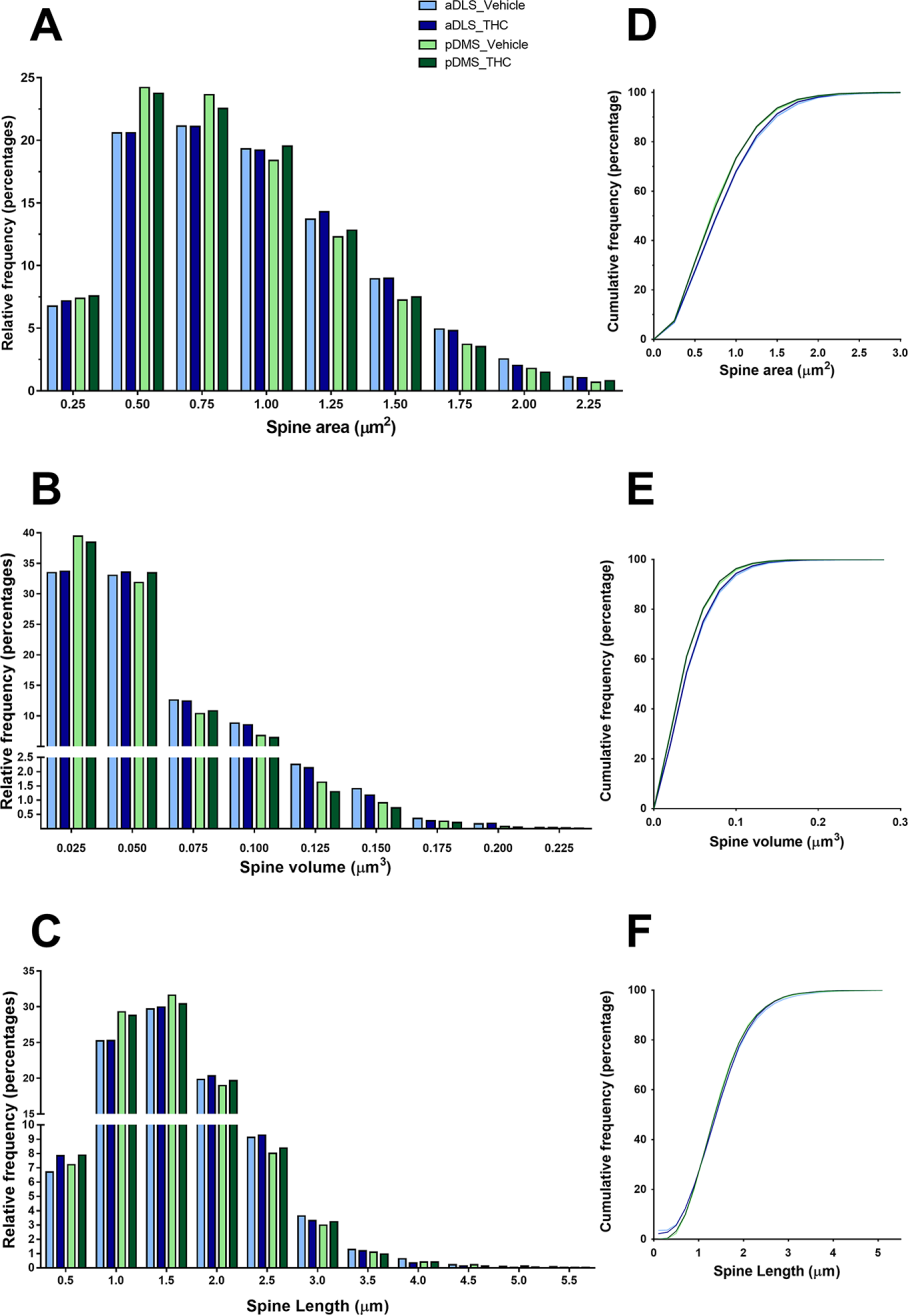
Frequency distribution of spine size parameters in the whole dendrite. The frequency distributions (A, B and C), and cumulative frequency distribution functions as percentages (D, E and F) of spine size parameters analysed. Spine area (A, D), spine volume (B, E), and spine length (C, F). Frequency distributions and cumulative frequencies are plotted as unsmoothed data considering all the spines in each group; n = 8 mice per group.

To further investigate spine size, we studied frequency and cumulative frequency distributions of spine volume, spine area, and spine length (Fig 8). The K–S two-sample test revealed an effect of *region* in both VH- and THC-treated animals. Compared to aDLS neurons, pDMS neurons had a higher percentage of small spines, with smaller area (D = 0.06872, p < 0.0001; D = 0.055, p < 0.0001 for VH and THC, respectively) and volume (D = 0.06989, p < 0.0001; D = 0.06343, p < 0.0001 for VH and THC, respectively). No differences in the frequency distribution for spine length were observed.

## Discussion

We have studied here the effects of prolonged THC treatment on dendritic and spine morphology of medium spiny neurons (MSNs) in the anterior dorsolateral striatum (aDLS) and posterior dorsomedial striatum (pDMS)—two regions which are thought to be involved in the transition from some forms of goal-oriented behaviour to habit learning (for a review see [15]). The most notable effects we found were the following: 1) extended THC administration was related to a selective increase in dendritic spine density in the distal part of pDMS dendrites, whereas no effects were observed in aDLS; 2) we observed region-specific differences between aDLS and pDMS MSN dendrites regarding dendritic spine areas and volumes; and 3) no differences in dendritic size were observed either as a consequence of THC treatment or between aDLS and pDMS neurons. We did not attempt to classify the spines into the five main groups of morphological types which are frequently recognized: stubby, mushroom, thin, filopodial and branched. In this regard, it should be noted that on the same dendrite there may be a continuum of spine shapes, and the morphology of a given spine can change rapidly through activity-dependent and -independent mechanisms [53–59]. Therefore, in the present study we focused on the general quantitative differences in spine volume, area, and length measurements.

It is important to note that the observed differences were compartment-specific, since THC treatment increased distal but not proximal or total dendritic spine density in pDMS. Moreover, lower spine volumes and surface areas were found in the pDMS compared to the aDLS, when either the whole dendrite or the distal parts of the dendrites were considered. Compartment-specific modifications were consistent with other studies that reported changes in spines after chronic exposure to other drugs of abuse [43,44]. As we mentioned previously, the inputs to the distal dendrites originate from the cerebral cortex, the nigrostriatal DA afferents and the thalamus [38]. CB1 receptors have been localized to presynaptic nerve terminals of glutamatergic corticostriatal projection neurons and GABAergic MSNs [60,61]. Thus, THC-induced effects on the distal part of the dendrites indicate that these alterations might be related to specific inputs to MSNs. This result is consistent with the reduction in dendritic spine number localized in second order dendritic branches of the shell region of the nucleus accumbens (Nacc) MSNs after withdrawal from chronic THC administration, where DA-containing terminals make synaptic contacts [62]. There are also electrophysiological studies which have shown a functional dichotomy in striatal MSNs, where distal spines have been associated with prolonged “up states” in the dendritic tips [42]. This functional feature of distal spines is also well documented in other brain regions such as the striatum, amygdala and cerebral cortex [63–66].

There is considerable evidence that drugs of abuse induced long-lasting whole cell plasticity changes as well as alterations in spine density and spine morphology in different regions of the brain (for review see [67]). In addition, it has been shown that such effects in the case of cocaine and nicotine are mediated by CB1 receptors [68,69]. Similar to other drugs, repeated exposure to cannabinoids has been associated with microanatomical changes in the ventral tegmental area, the hippocampus, Nacc and prefrontal cortex neurons [33–37]. In accordance with these studies, our results showed that prolonged THC administration induced selective structural changes in the striatal MSNs. One important point regarding the dynamics of the reported changes in the striatal compartments after THC administration should be pointed out: with the design that we have implemented in these experiments, it is not possible to determine whether the changes in spine density in distal dendrites are a result of the effects of THC per se or an adaptation to the withdrawal from the drug. Adding to this complication is the fact that THC remains stored in lipid tissues for long periods of time [70,71], making it even more difficult to tease these two effects apart, and this would have been the case even if an acute exposure group had been added. In the literature about the effects of cannabinoid on dendritic spines, studies typically examine long-term effects, and they rarely include an acute injection group or analyse the effects of the cannabinoid immediately after the treatment. A notable exception is the work by Spiga and collaborators [37,62]. These authors found no evidence for alterations in dendritic spine density in the Nacc (core or shell) after chronic THC (or the synthetic cannabinoid CP 55,940). However, after a short withdrawal period (1 hour) a decrease in spine density in the core was observed, after both spontaneous and antagonist-precipitated withdrawal. Another study by Carvalho and colleagues found that a chronic treatment with the cannabinoid agonist WIN decreased the spine density in the Nacc (the authors did not distinguish between core and shell) as early as 24 hours after the last injection [72]. Although we have analysed the dorsal striatum and not the Nacc, the dynamics of the reported changes might be expected to be similar. Considering all these pieces of evidence, we suggest that the changes that we have observed may emerge soon after the treatment and they would be long lasting. Indeed, it was previously reported that chronic exposure to THC modified the structure of the dendrites in the Nacc shell and the mPFC one month after THC cessation with lower doses than in the present study [34], and a more recent study reports that changes in spine density endures for 90 days in these regions [73].

We also observed region-specific differences. In this regard, pDMS neurons have lower spine volumes and surface areas than aDLS neurons. Changes in dendritic spine morphology seem to be important for behavioural plasticity [74,75] and have been correlated with synaptic strength (for a review see [76]). For example, it has been postulated that spines with small head diameters are unstable and responsible for the acquisition of memory and that large spines are more stable and necessary for long-term memory formation [57,77]. It has also been suggested that these larger spines may evolve from the maturation of the thinner spines [78]. Also, it has been proposed that only small spines can expand their head volume after they are activated, while the large ones do not change [79]. The fact that we found a larger percentage of small spines in the pDMS in comparison to the aDLS might suggest that the activation of small spines in the pDMS could facilitate modifications in their morphology, which could lead to greater synaptic flexibility in this area.

We did not find any changes in dendritic size as a consequence of THC treatment. This seems to be in agreement with another study where the effects of chronic THC on neural structure were examined; differences in dendrite length were found in Nacc shell and medial prefrontal cortex, but not in the dorsal striatum, hippocampus, orbital frontal cortex, parietal cortex, or occipital cortex [34]. Different studies which have analysed the effects of other abused drugs on dendrite size have found an increase in dendritic length, but again the effect was described in the cingulate cortex but not in the Nacc [80,81]. When such effects on dendrite size take place, they are far from subtle. In fact, in agreement with our results, most of the studies that evaluate the effects of drug abuse have found changes related to dendritic spine density and morphology [44,82–84]. In addition, we did not find differences between aDLS and pDMS in terms of dendritic volume or area. DLS MSN dendritic arbors were recently reported to be larger and more complex compared to DMS dendritic arbors [85], but this study did not assess exactly the differences between pDMS and aDLS neurons and was conducted in rats. It is important to note that in our study we focused on the morphological characteristic of the dendrites, but not on the neuronal structure. Therefore, the effects of THC on neuronal morphology in pDMS and aDLS and the underlying differences between these regions would need to be addressed.

It has been reported that the influence of cannabinoids on dorsal striatal synaptic plasticity is limited to the DLS, whereas the endocannabinoid system appears to play little or no role in synaptic plasticity within the DMS [46]. This was suggested to be attributed to the high expression of CB1 receptors in the DLS and the relatively low expression of CB1 receptors in the DMS. However, in the medial area of the rostral caudate–putamen, there were numerous intensely stained immunoreactive fibre bundles [86] and this fact could explain our observations regarding the effects of THC on dendritic spine in pDMS neurons.

Finally, we have observed that spines located at the proximal part of the dendrite were smaller than distal spines, regardless of the region or drug treatment—a fact that has also been reported in different regions and species: granule cells in the dentate gyrus, in the pyramidal cells of the primary visual cortex and CA1 hippocampal neurons of young adult mice and rats [87,88], as well as in the basal dendrites of pyramidal cells of the human cingulate cortex [89].

## Conclusions

Taken together, the results obtained in this study suggest that differences in the structure of pDMS and aDLS dendritic spines may be related to their functional specialization. Moreover, the alterations that prolonged THC administration induces in MSN dendritic spines in pDMS could be related to the influence of THC on habit formation.

## Supporting information

S1 Fig Individual datapoints for dendrite size parameters shown in Fig 2. (TIF)

S2 Fig Individual datapoints for dendritic spine density shown in Figs 3 and 4. (TIF)

S3 Fig Individual dataponits for spine size parameters shown in Figs 5–7. (TIF)

S1 File NC3Rs ARRIVE guidelines checklist.pdf. (PDF)
