# Supplementary figures and images for: Selective effects of Δ9-tetrahydrocannabinol on medium spiny neurons in the striatum
Source: PLoS One. 2018 Jul 26;13(7):e0200950. doi: 10.1371/journal.pone.0200950 (PMC6062058; doi:10.1371/journal.pone.0200950)

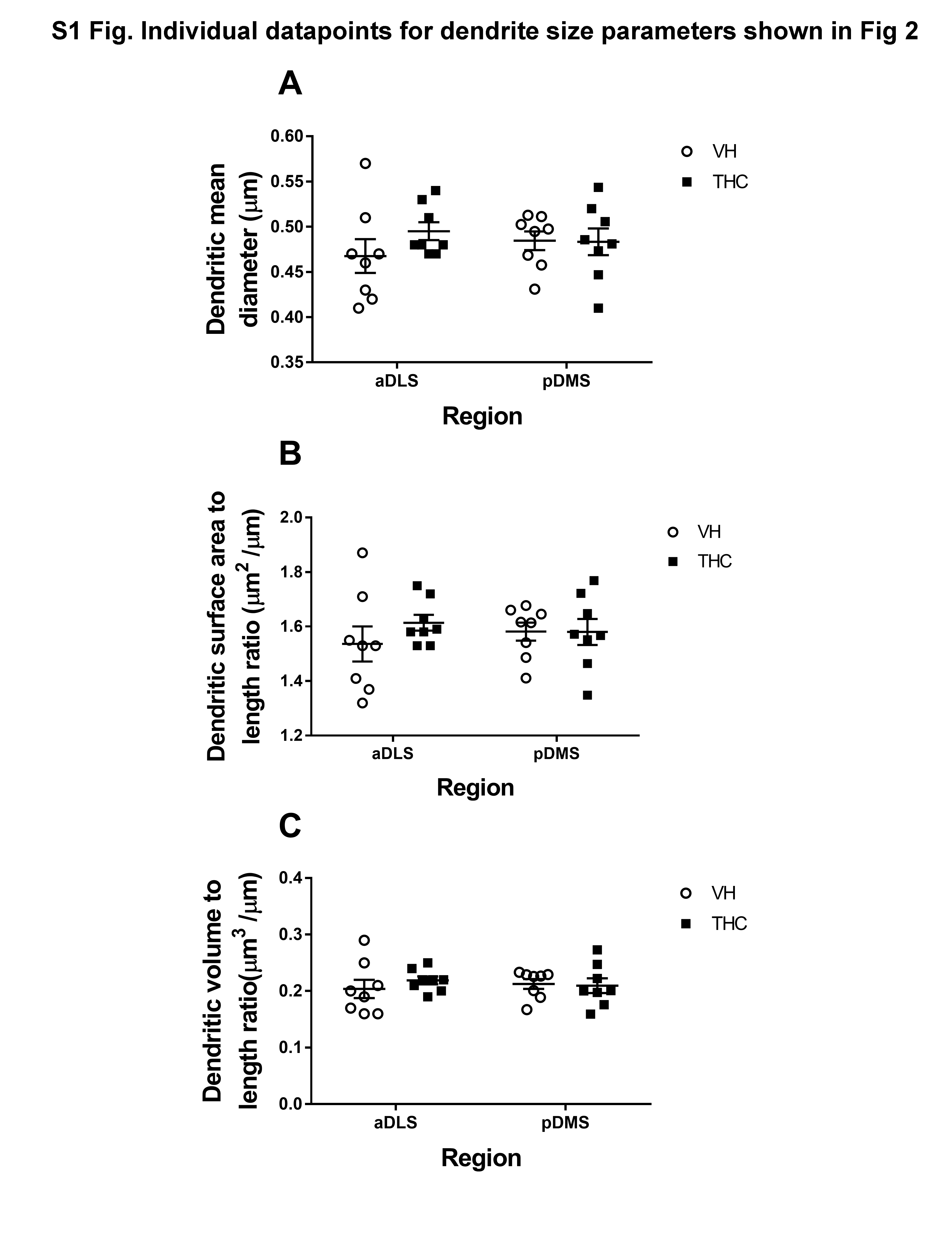

Supplement: S1 Fig — (TIF) [file pone.0200950.s001.tif]

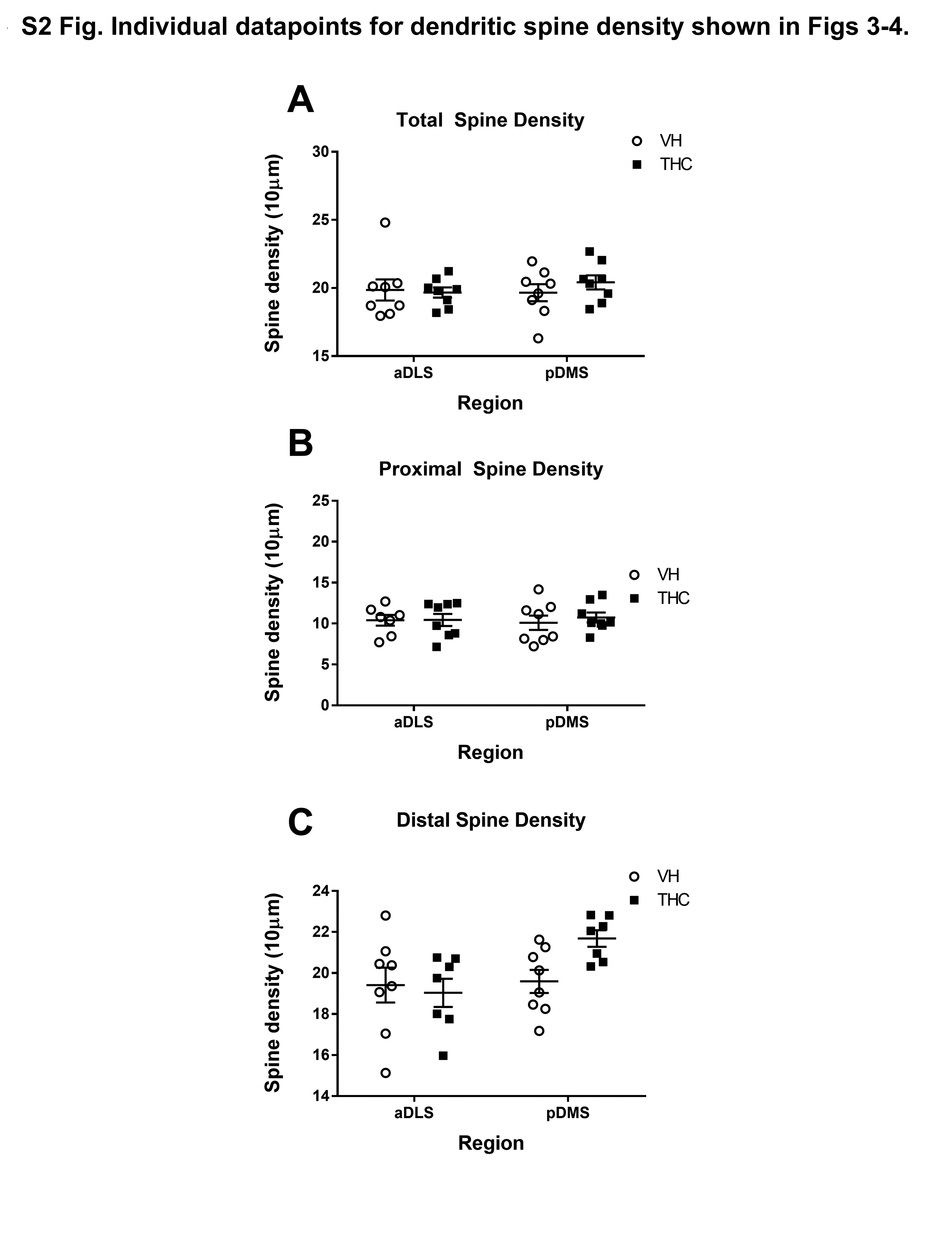

Supplement: S2 Fig — (TIF) [file pone.0200950.s002.tif]

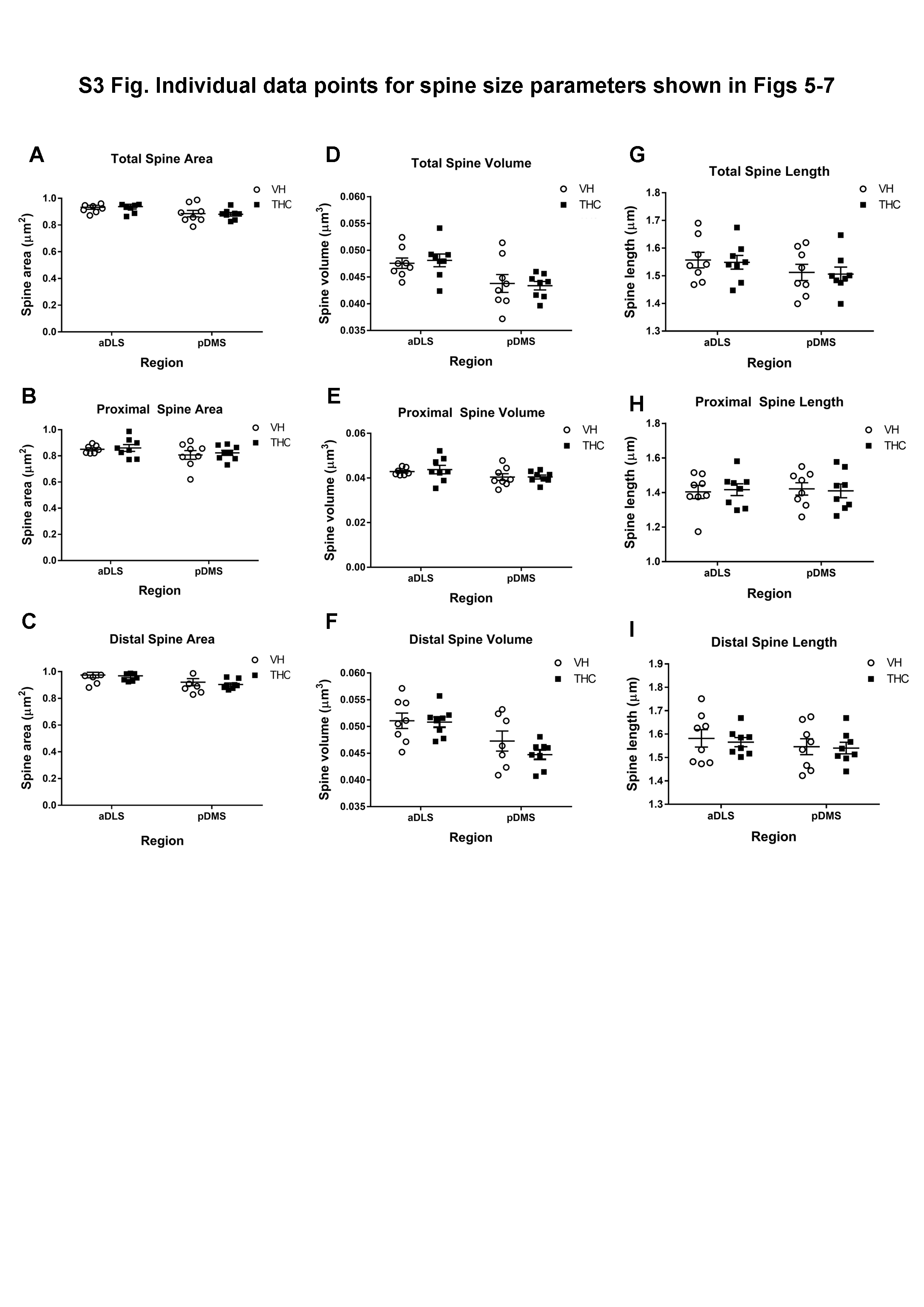

Supplement: S3 Fig — (TIF) [file pone.0200950.s003.tif]
